# Rapid decline of adaptation of *Pseudomonas fluorescens* to soil biotic environment

**DOI:** 10.1098/rsbl.2021.0593

**Published:** 2022-03-09

**Authors:** Pedro Gómez, Alex R. Hall, Steve Paterson, Angus Buckling

**Affiliations:** ^1^ Centre for Ecology and Conservation, School of Biosciences, University of Exeter, Cornwall Campus, Penryn TR10 9EZ, UK; ^2^ Department of Ecology, Evolution and Behaviour, University of Liverpool, Liverpool, UK

**Keywords:** bacteria fitness, cost of adaptation, microbial community, *Pseudomonas fluorescens*, potting soil

## Abstract

Interactions between microbes can both constrain and enhance their adaptation to the environment. However, most studies to date have employed simplified microbial communities and environmental conditions. We determined how the presence of a commercial potting compost microbial community affected adaptation of the soil bacterium *Pseudomonas fluorescens* SBW25 in potting compost. *Pseudomonas fluorescens* clones isolated from populations evolved in both the presence and absence of the community showed similar fitness increases when measured in the absence of the community. This suggests the presence of the community did not constrain adaptation. By contrast, fitness measured in the presence of the community increased for community-evolved populations, but decreased below the ancestral state for populations evolved in the absence of the community. This suggests some, but not all, mutations that were beneficial with respect to the abiotic environment were costly in the presence of the community, with the former selected against in the presence of the community. Whole-genome sequencing supports this interpretation: most mutations underpinning fitness changes were clone-specific, suggesting multiple genetic pathways to adaptation. Such extreme mutational effects have not been observed in comparable *in vitro* studies, suggesting that caution is needed when extrapolating results from simplified *in vitro* systems to natural contexts.

## 1. Introduction

Understanding how the presence of interacting species affects adaptation to other (abiotic and biotic) components of the environment is a fundamental aspect of evolutionary ecology [1]. Adaptation may be constrained by other species through a range of processes, including reductions in population size (in the case of negative interactions) [2], trade-offs between adaptation to different components of the environment [3] and species filling ecological niches faster than evolution occurs [4,5]. Conversely, adaptation may be enhanced if interactions increase population size, open up new ecological niches [6,7] or buffer population sizes in the face of environmental perturbations [8]. Thus, examining the extent to which community affects species interactions is crucial to understand how species adapt and shape ecosystem functioning.

Experimental work in this area has primarily used microbes, because of the speed at which they evolve. Studies frequently report constrained adaptation to other components of the environment as a consequence of species interactions, primarily through reductions in population size and trade-offs [3,9–13] (but see [14]). However, the majority of studies focus on short-term adaptation to greatly simplified communities grown under nutrient-rich *in vitro* conditions. The novelty of the abiotic environments and the high densities of other organisms will invariably impose very strong selection on the focal organism, potentially leading to findings that may not be observed under more natural conditions. Even in conditions where the abiotic environment more closely resembles a natural environment (such as the use of beech leaf ‘tea’, to emulate a beech tree hole environment [10], or wheat grass [14]), the interacting community is invariably greatly simplified.

In this study, we examine the interplay between adaptation to the biotic and abiotic environment within a managed natural system: potting compost. We previously evolved the soil and plant-associated bacterium *Pseudomonas fluorescens* SBW25 in commercial potting compost, in the presence or absence of the naturally associated community [15,16]. We found that the community imposed selection on the population (density was reduced) and inhibited metabolic diversification [16]. Here, we measure the fitness of evolved clones in the presence and absence of the resident microbial community by competing each against an isogenic marked ancestral strain. Additionally, we sequence whole genomes of the clones in an attempt to provide insight into mechanisms underpinning fitness differences.

## 2. Material and methods

### (a) Selection experiment

From a previous study [15], we randomly isolated single bacterial clones of a gentamicin-resistant strain of *P. fluorescens* SBW25 [17] that had been evolving in 12 independent populations in commercial potting compost (John Innes no. 2) microcosms for 48 days; six in the presence and six in the absence of the natural potting compost community. Briefly, 5 ml of a *P. fluorescens* suspension (at 2 × 10^8^ CFU ml^−1^ in M9 buffer) was inoculated into 12 polypropylene trays (10 × 10 cm) with lids containing 100 g of twice-autoclaved potting compost. The potting compost microbial community from a potting compost wash (20 g of potting compost in 100 ml M9 buffer [15,16,18,19]) was inoculated into half of the microcosms. Microcosms were placed in an environmental chamber at 26°C and 80% relative humidity. After 48 days, a soil suspension wash from each of the 12 microcosms was plated onto gentamicin (15 µg ml^−1^) KB agar plates, and individual clones isolated. Note that the previous experiment focused on bacteria–phage coevolution, but here we only focus on the phage-free control populations.

### (b) Competition assays

Competition experiments between all bacterial clones and a *lacZ*-marked SBW25 ancestor were carried out as in previous studies [20,21] to estimate the fitness of evolved bacteria in both the presence and absence of the soil microbial community. Briefly, bacterial clones were independently grown in Lysogeny Broth (LB) liquid medium overnight, and 5 ml M9 buffer (minimal salts solution) containing approximately 10^8^ CFU of each clone was inoculated into two microcosms each, along with the same density and volume of the ancestral competitor. The soil microbial community, or M9 buffer only, was then added to one of the microcosms per clone. Prior to inoculation and after 5 days growth, bacterial population densities were determined by plating on LB agar supplemented with X-gal (40 µg ml^−1^), in order to distinguish *lacZ-*marked *P. fluorescens* SBW25 strain and evolved SBW25 populations [20,22]. Selection rate constants (*S* = *m*_evolved_ − *m*_ancestor_, where *m* = ln (density after 5 days/starting density)) [23] were calculated for each clone; positive values of *S* indicate higher fitness of the evolved bacteria as compared with the ancestor. Competition experiments were replicated three times per clone.

### (c) Genome re-sequencing

The whole genomes of the 12 bacterial clones were sequenced by HiSeq-Illumina technology at the Centre for Genomic Research (University of Liverpool). First, each bacterial clone was incubated at 28°C and shaking at 140 r.p.m. overnight, reaching densities of approximately 10^9^ CFU ml^−1^. Then bacterial cultures were aliquoted to carry out the total genomic DNA extraction, which was performed using the Qiagen DNeasy Blood and Tissue kit according to the manufacturer's instructions. DNA libraries were prepared with the Illumina-TrueSeq kit and sequenced by 2 × 100 bp paired-end reads on an Illumina-HiSeq2000 platform. Casava v. 1.8.2, Cutadapt v. 1.2.2 and Sickle v. 1.200 were used to perform the basecalling, de-multiplexing and trimming of the indexed reads, with a minimum window quality score of 20, and reads with more than 3 bp of adapter or shorter than 10 bp were removed. Per sample, an average of 12.4 million filtered read pairs (range 7.3–18.7 million) were mapped to the SBW25 reference genome (GenBank NC_012660.1) using BWA (v. 0.5.9-r16), with local realignment and variant calling (relative to the ancestral SBW25 genome sequenced at the same time) achieved using GATK Unified Genotyper (v. 2.1-13-g1706365) followed by snpEff (v. 4.1) to assign effects on coding genes. Only non-synonymous SNPs with high impact effect were considered. The data for this study have been deposited in the European Nucleotide Archive (ENA) at EMBL-EBI under accession number PRJEB38430.

### (d) Data analyses

Relative fitness analysis for each individual bacterial clone was performed with a linear mixed effects model fitted with REML, where the selective environment (presence and absence of the community) was fitted as a main factor, and nested replicates (*n* = 3) as a random effect. This was carried out in JMP software. To test whether different sets of genes were mutated in clones evolved in the absence/presence of the community, we used permutational analysis of variance, PERMANOVA [24,25], using the adonis function of the vegan package in R v. 3.3.3 and Euclidean distance as the measure of dissimilarity (distance was measured at the level of mutated genes, so the distance between two clones decreases if they have mutations in the same genes, even if the nucleotide changes involved are different). Dataset files are available from the Dryad repository linked to https://doi.org/10.5061/dryad.vdncjs [26].

## 3. Results

### (a) Fitness of *P. fluorescens* clones in the presence and absence of the community

**Figure 1.**
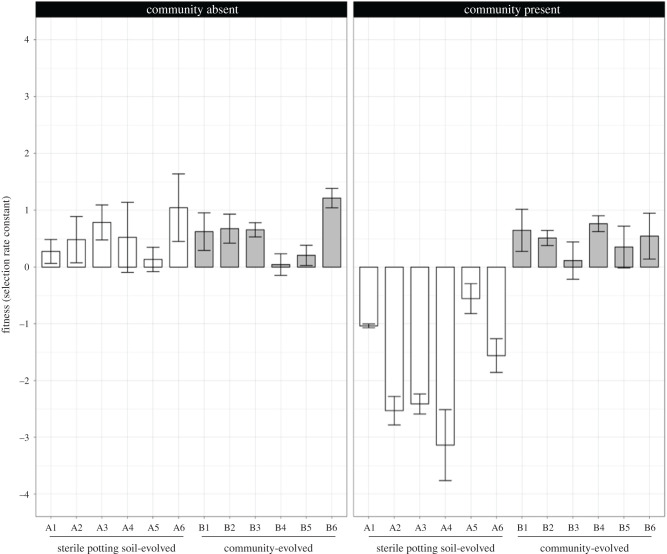
Fitness (selection rate constant) of *P. fluorescens* SBW25 in different selective environments: in sterile potting soil and in potting soil with the microbial community. Each bar represents the bacterial growth rate of the different evolved clones (A: in the absence, and B: in the presence of the microbial community) related to the ancestral after 5 days competition calculated by the difference in the estimated Malthusian parameter (*m*). Each competition assay was performed with three replicates. Positive values indicate higher relative fitness of the evolved bacteria as compared with the ancestor.

We measured the fitness of each bacterial clone in both selective environments (i.e. in the presence and absence of the microbial community), and found that there was a significant interaction between the selection environment and the environment in which fitness was measured (*F*_1,58_ = 82.32, *p* < 0.001). Figure 1 shows that bacterial clones that were evolved in sterile potting compost or in the presence of the community had similar fitness in the absence of community (*F*_1,10_ = 0.018, *p* = 0.896), with fitness greater than the ancestor in both treatments (*t*_5_ = 4.83 and *t*_5_ = 5.64; *p* < 0.002). However, community-evolved populations had much greater fitness in the presence of the community than populations evolved in sterile potting compost only (*F*_1,10_ = 32.64, *p* < 0.002), with the former showing significantly higher fitness than the ancestor (*t*_5_ = 6.12, *p* < 0.001), and the latter significantly lower (*t*_5_ = 8.22, *p* < 0.001). The community-evolved populations had approximately equal fitness in both environments (*t*_11_ = 0.63, *p* < 0.533). These data suggest no cost to adaptation to the community, while populations rapidly became maladapted to the community when evolved in its absence.

### (b) Genetic changes in *P. fluorescens* bacteria clones

We re-sequenced the 12 evolved clones to attempt to identify mutations underpinning the phenotypic differences between treatments. The number of non-synonymous single nucleotide polymorphisms (SNPs) ranged between 0 and 17 per clone (table 1), and between 1 and 6 INDELs (table 2), with mean numbers of each not differing between treatments (Welch's *t*-test: *p* > 0.05 for SNPs and INDELs). The majority of mutations were unique to individual clones. There were seven cases (4 SNPs and 3 INDELs) where the same gene was mutated in two out of six clones in one treatment and zero out of six in another; six of these seven genes were mutated only in clones evolved without the community. Despite this, clones evolved in the same treatment group (with/without community) did not have significantly smaller genetic distances than those from different treatment groups (PERMANOVA at level of SNPs: *F*_1,10_ = 1.15, *p* = 0.18; INDELs: *F*_1,10_ = 1.17, *p* = 0.33; SNPs and INDELS combined: *F*_1,10_ = 1.14, *p* = 0.21). Linking mutated genes to specific biological process (tables 1 and 2) did not reveal any pattern between treatments in the functional consequence of mutations. For example, *PFLU2423* and *PFLU3233* are both components of type II secretion and both only mutated in absence-evolved, but *PFLU3230* is also linked to type II SS and mutated in presence. One gene (*PFLU1668*—a putative epimerase) was mutated in three populations evolved with the community and two without. In particular, the A2 and B1 clones displayed the similar insertion type at the same location for this *PFLU1668* gene, possibly generating the same alternative sequence, but it should be noted that the A2 clone additionally exhibited a non-synonymous mutation in the *PFLU2493* hypothetical gene predicted to be of moderate impact. Taken together, these results suggest there are many mutations that can lead to adaptation and maladaptation to the complex potting soil environment.

**Table 1.** Genetic characterization for the evolved clones of *P. fluorescens* SBW25 (A: in the absence, and B: in the presence of the microbial community). The occurrence of non-synonymous single nucleotide polymorphisms (SNPs) is marked in binary format (0: absence, 1: presence) after filtering with a cut-off of 95% frequency. The reference of each gene, in addition to the subcellular localization and the biological processes are provided along with the end-product affected by functional categories.

*P. fluorescens* SBW25 clone																
A						B										
1	2	3	4	5	6	1	2	3	4	5	6	gene	product name	subcelullar localization	biological process (GO term)	functional category
1	0	1	0	0	0	0	0	0	0	0	0	*PFLU0988*	putative alginate biosynthesis-like protein	periplasmic	efflux pump membrane protein (multidrug resistance protein A)	drug transport
1	0	1	0	0	0	0	0	0	0	0	0	*PFLU1511*	putative transporter-like membrane protein	cytoplasmic membrane	response to drug	drug binding
1	0	1	0	0	0	0	0	0	0	0	0	*PFLU3233*	putative general secretory pathway protein	unknown	type II secretory pathway, component PulM	transport
1	0	0	0	1	0	0	0	0	0	0	0	*ureC*	urease subunit alpha	cytoplasmic	urea metabolic process	resource utilization
1	0	0	0	0	0	0	0	0	0	0	0	*gltP*	glutamate/aspartate:proton symporter	cytoplasmic membrane	Na^+^/H^+^-dicarboxylate symporters	transport
1	0	0	0	0	0	0	0	0	0	0	0	*lepB*	signal peptidase I	cytoplasmic membrane	protein secretion	resource utilization
1	0	0	0	0	0	0	0	0	0	0	0	*PFLU0808*	putative transporter-like membrane protein	cytoplasmic membrane	sulfite reductase complex (NADPH)	cellular component
1	0	0	0	0	0	0	0	0	0	0	0	*PFLU1694*	putative ABC transporter ATP-binding protein	cytoplasmic membrane	ABC-type multidrug transport system, ATPase component	resource utilization
1	0	0	0	0	0	0	0	0	0	0	0	*PFLU1829*	hypothetical protein	unknown	PaaI_thioesterase	catalytic activity
1	0	0	0	0	0	0	0	0	0	0	0	*PFLU2096*	putative transporter-like membrane protein	cytoplasmic membrane	arabinose efflux permease	resource utilization
1	0	0	0	0	0	0	0	0	0	0	0	*PFLU2490*	putative chloride transport channel membrane protein	cytoplasmic membrane	chloride channel protein EriC	membrane component
1	0	0	0	0	0	0	0	0	0	0	0	*PFLU2809*	UDP pyrophosphate phosphatase	cytoplasmic membrane	undecaprenyl-diphosphatase activity	unknown
1	0	0	0	0	0	0	0	0	0	0	0	*PFLU2923*	AraC family transcriptional regulator	cytoplasmic	AraC-type DNA-binding domain-containing proteins	regulation biological process
1	0	0	0	0	0	0	0	0	0	0	0	*PFLU2961*	arsenical pump membrane protein	cytoplasmic membrane	response to arsenic-containing substance	response to stimulus
1	0	0	0	0	0	0	0	0	0	0	0	*PFLU4512*	putative transmembrane transport protein	cytoplasmic membrane	purine-cytosine permease and related proteins	catalytic activity
1	0	0	0	0	0	0	0	0	0	0	0	*PFLU4789*	putative glycerophosphoryl diester phosphodiesterase	periplasmic	glycerophosphoryl diester phosphodiesterase	catalytic activity
1	0	0	0	0	0	0	0	0	0	0	0	*rpoN*	RNA polymerase factor sigma-54	cytoplasmic	sigma factor activity	resource utilization
0	0	0	1	0	0	0	0	0	0	0	0	*PFLU0584*	putative amino acid ABC transporter ATP-binding protein	cytoplasmic membrane	ATP-binding cassette (ABC) transporter complex, substrate-binding subunit-containing	membrane component
0	0	0	1	0	0	0	0	0	0	0	0	*PFLU1686*	putative RHS repeat-like protein	outer membrane	Rhs family protein	cellular component
0	0	0	1	0	0	0	0	0	0	0	0	*PFLU3083*	putative dioxygenase	cytoplasmic	catechol 2,3-dioxygenase activity	catalytic activity
0	0	0	1	0	0	0	0	0	0	0	0	*PFLU5328*	hypothetical protein	cytoplasmic membrane	putative threonine efflux protein	transport
0	0	0	1	0	0	0	0	0	0	0	0	*PFLU5329*	putative sensory box GGDEF/EAL domain-containing protein	cytoplasmic membrane	signal transduction	regulation biological process
0	0	0	1	0	0	0	0	0	0	0	0	*potF1*	putrescine ABC transporter substrate-binding periplasmic protein	periplasmic	ATP-binding cassette (ABC) transporter complex, substrate-binding subunit-containing	membrane component
0	0	0	1	0	0	0	0	0	0	0	0	*recG*	ATP-dependent DNA helicase RecG	cytoplasmic	DNA recombination	DNA recombination
0	0	0	0	1	0	0	0	0	0	0	0	*PFLU0052*	putative dehydrogenase	unknown	choline dehydrogenase and related flavoproteins	catalytic activity
0	0	0	0	1	0	0	0	0	0	0	0	*PFLU0310*	hypothetical protein	cytoplasmic	unknown	unknown
0	0	0	0	1	0	0	0	0	0	0	0	*PFLU0458*	hypothetical protein	cytoplasmic membrane	cyclase activity	catalytic activity
0	0	0	0	1	0	0	0	0	0	0	0	*PFLU1581*	hypothetical protein	outer membrane	surface lipoprotein	transport
0	0	0	0	1	0	0	0	0	0	0	0	*PFLU2489*	hypothetical protein	cytoplasmic	methylisocitrate lyase activity	catalytic activity
0	0	0	0	1	0	0	0	0	0	0	0	*PFLU2753*	putative EAL/GGDEF domain-containing signalling protein	cytoplasmic membrane	cyclase activity	catalytic activity
0	0	0	0	1	0	0	0	0	0	0	0	*PFLU3370*	hypothetical protein	cytoplasmic membrane	oxidoreductase activity	metabolic process
0	0	0	0	1	0	0	0	0	0	0	0	*PFLU3508*	hypothetical protein	cytoplasmic membrane	unknown	unknown
0	0	0	0	1	0	0	0	0	0	0	0	*PFLU3940*	allantoate amidohydrolase	cytoplasmic	acetylornithine deacetylase/succinyl-diaminopimelate desuccinylase and related deacylases	catalytic activity
0	0	0	0	1	0	0	0	0	0	0	0	*PFLU3948*	putative family S43 non-peptidase protein	outer membrane	unknown	unknown
0	0	0	0	1	0	0	0	0	0	0	0	*PFLU4304*	LysR family transcriptional regulator	cytoplasmic	transcriptional regulator	regulation biological process
0	0	0	0	1	0	0	0	0	0	0	0	*PFLU5618*	hypothetical protein	unknown	predicted phosphatase	resource utilization
0	0	0	0	0	0	0	1	0	0	0	0	*cyaA*	adenylate cyclase	cytoplasmic membrane	adenylate cyclase	resource utilization
0	0	0	0	0	0	0	1	0	0	0	0	*PFLU0916*	putative methyl-accepting chemotaxis protein	cytoplasmic membrane	methyl-accepting chemotaxis protein	catalytic activity
0	0	0	0	0	0	0	1	0	0	0	0	*PFLU2428*	hypothetical protein	extracellular	unknown	unknown
0	0	0	0	0	0	0	1	0	0	0	0	*PFLU2600*	cyn operon positive regulator	cytoplasmic	transcriptional regulator	regulation biological process
0	0	0	0	0	0	0	1	0	0	0	0	*PFLU2693*	putative haloacid dehalogenase-like hydrolase	unknown	hydrolase activity	catalytic activity
0	0	0	0	0	0	0	1	0	0	0	0	*PFLU3596*	hypothetical protein	outer membrane	unknown	unknown
0	0	0	0	0	0	0	1	0	0	0	0	*phnH*	carbon-phosphorus lyase complex subunit	cytoplasmic	uncharacterized enzyme of phosphonate metabolism	unknown
0	0	0	0	0	0	0	1	0	0	0	0	*pobA*	4-hydroxybenzoate 3-monooxygenase	cytoplasmic	benzoate metabolic process	resource utilization
0	0	0	0	0	0	0	0	1	0	0	0	*PFLU0850*	putative aldehyde dehydrogenase	cytoplasmic	betaine-aldehyde dehydrogenase activity	catalytic activity
0	0	0	0	0	0	0	0	0	0	1	0	*PFLU3230*	general secretion pathway protein F/S	cytoplasmic membrane	protein secretion by the type II secretion system	resource utilization
0	0	0	0	0	0	0	0	0	0	1	0	*PFLU3585*	putative FAD-dependent oxidoreductase	cytoplasmic	glycine/d-amino acid oxidases (deamination)	catalytic activity
0	0	0	0	0	0	0	0	0	0	1	0	*PFLU3747*	putative sigma-54-activated regulatory protein	cytoplasmic	transcriptional activator of acetoin/glycerol metabolism	resource utilization
0	0	0	0	0	0	0	0	0	0	1	0	*PFLU3920*	2-oxoacid dehydrogenase subunit E1	cytoplasmic	cytosolic pyruvate dehydrogenase complex	cellular component
0	0	0	0	0	0	0	0	0	0	1	0	*PFLU4306*	putative GGDEF/GAF domain sensory box protein	cytoplasmic	cyclase activity	catalytic activity
0	0	0	0	0	0	0	0	0	0	1	0	*PFLU4516*	LysR family transcriptional regulator	cytoplasmic	transcriptional regulator	regulation biological process
0	0	0	0	0	0	0	0	0	0	0	1	*ddl*	d-alanyl-alanine synthetase A	cytoplasmic	peptidoglycan biosynthetic process	resource utilization
0	0	0	0	0	0	0	0	0	0	0	1	*PFLU0087*	putative two-component system sensor kinase	cytoplasmic membrane	phosphate ion transport	transport
0	0	0	0	0	0	0	0	0	0	0	1	*PFLU0478*	putative glycosyl transferase	cytoplasmic	glycosyltransferase	catalytic activity
0	0	0	0	0	0	0	0	0	0	0	1	*PFLU1394*	putative hydrolase	unknown	beta-lactamase class C and other penicillin-binding proteins	drug binding
0	0	0	0	0	0	0	0	0	0	0	1	*PFLU3262*	putative amidase	cytoplasmic	glutaminyl-tRNA synthase (glutamine-hydrolysing) activity	catalytic activity
0	0	0	0	0	0	0	0	0	0	0	1	*PFLU3345*	hypothetical protein	unknown	unknown	unknown
0	0	0	0	0	0	0	0	0	0	0	1	*PFLU3969*	putative acetyltransferase	unknown	ribosome biogenesis	cellular component
0	0	0	0	0	0	0	0	0	0	0	1	*PFLU6085*	putative cobalamin biosynthesis-like protein	cytoplasmic	cobalamin biosynthetic process	catalytic activity
0	0	0	0	0	0	0	0	0	0	0	1	*prfA*	peptide chain release factor 1	cytoplasmic	translational termination	resource utilization

**Table 2.** Genetic characterization for the evolved clones of *P. fluorescens* SBW25 (A: in the absence, and B: in the presence of the microbial community). The occurrence of indels (insertion or deletion) is marked in binary format (0: absence, 1: presence) after filtering with a cut-off of 95% frequency. The reference of each gene, in addition to the subcellular localization and the biological processes are provided along with the end-product affected by functional categories.

*P. fluorescens* SBW25 clone																
A							B									
1	2	3	4	5	6	1	2	3	4	5	6	gene	product name	subcelullar localization	biological process (GO term)	functional category
1	0	1	0	0	0	0	0	0	0	0	0	*PFLU2423*	putative type II secretion pathway protein D	outer membrane	type II secretory pathway, component PulD	cell motility
1	0	1	0	0	0	0	0	0	0	0	0	*PFLU3208*	amidase	cytoplasmic	Asp-tRNAAsn/Glu-tRNAGln amidotransferase A subunit and related amidases	translation, ribosomal structure and biogenesis
1	0	0	0	0	0	0	0	0	0	0	0	*PFLU3658*	putative extracellular polysaccharide biosynthesis protein	cytoplasmic membrane	sugar transferases involved in lipopolysaccharide synthesis	cell wall/membrane/envelope biogenesis
1	0	0	0	0	0	0	0	0	0	0	0	*PFLU0788*	zinc-binding protein	unknown	uncharacterized protein conserved	unknown
0	0	0	1	0	0	0	0	0	0	0	0	*PFLU1420*	LysR family transcriptional regulator	cytoplasmic	transcriptional regulator	transcription
0	0	0	0	1	0	0	0	0	0	0	0	*PFLU5282A*	putative DNA invertase	cytoplasmic	site-specific recombinases, DNA invertase Pin homologues	replication, recombination and repair
0	0	0	0	1	0	0	0	0	0	0	0	*codA*	cytosine deaminase	cytoplasmic	cytosine deaminase and related metal-dependent hydrolases	resource utilization
0	0	0	0	1	0	0	0	0	0	0	0	*PFLU2169*	putative LuxR family regulatory protein	cytoplasmic	response regulator containing a CheY-like receiver domain and an HTH DNA-binding domain	transcription
0	1	0	0	0	1	1	0	1	1	0	0	*PFLU1668*	putative polysaccharide biosynthesis-related membrane protein	cytoplasmic membrane	predicted nucleoside-diphosphate sugar epimerases	resource utilization
0	0	0	0	0	0	0	1	0	0	0	0	*PFLU1693*	putative ABC transporter membrane protein	cytoplasmic membrane	unknown	unknown
0	0	0	0	0	0	0	0	1	0	0	0	*PFLU0066*	protohaem IX farnesyltransferase	cytoplasmic membrane	polyprenyltransferase	post-translational modification
0	0	0	0	0	0	0	0	1	0	0	0	*PFLU3067*	3-oxoacyl-ACP reductase	cytoplasmic	dehydrogenases	resource utilization
0	0	0	0	0	0	0	0	1	0	0	0	*PFLU3323*	putative amino acid permease membrane protein	cytoplasmic membrane	amino acid transporters	resource utilization
0	0	0	0	0	0	0	0	1	0	0	0	*PFLU3510*	putative transmembrane protein	cytoplasmic membrane	unknown	unknown
0	0	0	0	0	0	0	0	1	1	0	0	*PFLU2381*	hypothetical protein	cytoplasmic membrane	arabinose efflux permease	resource utilization
0	0	0	0	0	0	0	0	0	0	1	0	*mutL*	DNA mismatch repair protein	cytoplasmic	DNA mismatch repair enzyme	replication, recombination and repair
0	0	0	0	0	0	0	0	0	0	1	0	*PFLU5103*	hypothetical protein	unknown	unknown	unknown
0	0	0	0	0	0	0	0	0	0	0	1	*PFLU0808*	putative transporter-like membrane protein	cytoplasmic membrane	sulfite reductase complex (NADPH)	resource utilization
0	0	0	0	0	0	0	0	0	0	0	1	*PFLU4038*	putative tartrate dehydrogenase	cytoplasmic	isocitrate/isopropylmalate dehydrogenase	resource utilization
0	0	0	0	0	0	0	0	0	0	0	1	*PFLU2281*	putative ABC transporter membrane protein	cytoplasmic membrane	ABC-type dipeptide/permease	resource utilization
0	0	0	0	0	0	0	0	0	0	0	1	*PFLU1849*	putative two-component system sensor kinase	cytoplasmic membrane	signal transduction histidine kinase	signal transduction mechanisms
0	0	0	0	0	0	0	0	0	0	0	1	*PFLU5027*	hypothetical protein	cytoplasmic membrane	unknown	unknown
0	0	0	0	0	0	0	0	0	0	0	1	*PFLU4348*	hypothetical protein	cytoplasmic membrane	unknown	unknown

## 4. Discussion

We investigated how the presence of the microbial community affected the rate of adaptation of a focal bacterium in a commercial potting compost. Populations that had been evolved for 48 days in both the presence and absence of a potting soil community showed equal increases in fitness when measured in the absence of the community. This suggests no major community-imposed constraint on adaptation, despite reductions in population size reported in our previous study [16]. This contrasts with many *in vitro* studies using highly simplified communities in nutrient media, where biotic interactions typically constrain adaptation. It is notable that in another recent study using a more natural environment, the community interactions increased abiotic adaptation of one of the species [14]. It is possible that the extreme selection pressures associated with laboratory environments may greatly exaggerate inhibitory effects of community interactions. Fitness increases of the community-evolved populations were comparable in both the presence and absence of the community, suggesting that most adaptation is to the abiotic environment. We previously reported increased metabolic diversity evolved in the absence of the community [16], but this clearly had no major effect on mean fitness of individual clones.

Our most striking finding, and not observed in comparable *in vitro* studies, is the large reduction in fitness in the presence of the community, following evolution in the absence. This suggests that some mutations (or epistatic combinations) confer advantages in the absence of the community, but are costly in the presence, i.e. they are antagonistically pleiotropic [27]. However, the absence of any obvious cost to adaptation of the community-evolved populations suggests that other equally accessible mutations are not antagonistically pleiotropic in these contexts. Our genomics analyses are consistent with this interpretation. Total mutations, including SNPs and INDELs, varied between 1 and 21 per clone, and the vast majority were unique. This suggests there are many ways in which populations could adapt to the complex potting soil environment. However, we note that by phenotyping and sequencing only a single clone per population, this between-population variation may be exaggerated given within-population variation. Mutations that were beneficial in the absence but costly in the presence of the community would be selected against when the community was present, and selected for in the absence (mutation accumulation [28]). Our results highlight the need to be cautious about extrapolating results from simplified *in vitro* systems to real-world contexts, particularly without clear-cut theoretical expectations.

## Data Availability

Dataset files are available from the Dryad repository, linked to https://doi.org/10.5061/dryad.vdncjs [26]. Sequencing data for this study have been deposited in the European Nucleotide Archive (ENA) at EMBL-EBI under accession number PRJEB38430.
